# In Vitro Acaricidal Activity of *Croton macrostachyus* Leaf and *Ricinus communis* Seed Extracts Against Cattle‐Infesting Ticks (*Rhipicephalus* spp. and *Amblyomma* spp.)

**DOI:** 10.1155/vmi/1189650

**Published:** 2026-02-23

**Authors:** Tesfaye Fatalo, Gebeyehu Alkadir

**Affiliations:** ^1^ Areka Agricultural Research Center, South Ethiopia Agricultural Research Institute, Dilla, Ethiopia; ^2^ College of Veterinary Medicine and Agriculture, Addis Ababa University, Bishoftu, Ethiopia, aau.edu.et

**Keywords:** Acaricidal efficacy, ambyloma, croton, in vitro, rhipicephalus, ricinus

## Abstract

Ticks are major ectoparasites of cattle and vectors of zoonotic and livestock diseases. Reliance on synthetic acaricides like diazinon has led to environmental concerns and rising resistance, prompting the search for ecofriendly alternatives. This in vitro study evaluated the acaricidal activity of *Croton macrostachyus*, *Ricinus communis*, and their combined extracts against *Rhipicephalus* and *Amblyomma* ticks. Adult ticks were exposed to serially diluted concentrations (125, 250, 500, and 1000 μg/mL) of plant extracts. Dimethyl sulfoxide and 0.1% diazinon served as negative and positive controls, respectively. Triplicate independent replication sets were performed. Both plant extracts exhibited significant (*p* < 0.05), dose‐ and time‐dependent mortality. *Croton macrostachyus* showed higher activity against *Amblyomma* (66.7 ± 1.53%) than *Rhipicephalus* (63.3 ± 1.16%) at 1000 μg/mL, while *Ricinus communis* conferred higher activity, achieving 80.0 ± 1.00% (*Amblyomma*) and 73.3 ± 0.58% (*Rhipicephalus*) mortality‐outperforming diazinon (70.0 ± 1.0%). Notably, the combined extract demonstrated synergistic effects with the highest mortality (83.3 ± 0.58%), indicating enhanced activity over the commercial acaricide. The moderate performance of diazinon supports concerns over acaricide resistance in the study area. Further in vivo trials and toxicity evaluations are essential before declaring the extracts as antitick.

## 1. Introduction

Ticks pose a significant risk to the health of animals and have considerable economic implications globally by causing irritation, skin infections, anemia, and tick fever and acting as vectors for various pathogens causing diseases [1]. The economic losses due to tick infestations on livestock contributing to food insecurity with global control costs and productivity losses are estimated at $7 billion USD annually [2]. Conventional tick control depends excessively on acaricides, contributing to ecosystem contamination, food safety risks, and escalating resistance undermining sustainability and increasing economic burdens [3].

Ethiopia’s reliance on synthetic acaricides for tick control has resulted in widespread resistance, environmental pollution, and food contamination due to inconsistent dosing and frequent misuse [4]. This has intensified the need for alternative approaches, making herbal medications an increasingly appealing solution that has gained significant importance in tropical and subtropical regions, particularly across Africa and Asia [5].

*Ricinus communis* seeds are rich in macromolecular components, primarily fixed oils containing glycosides of ricinoleic, isoricinoleic, stearic, and dihydroxystearic acids, while its dried leaves contain diverse bioactive compounds including the disaccharide glycoside rutin, flavonoids, phenolic acids, and tannins [6]. Phytochemical analysis of *Croton macrostachyus* has isolated diverse secondary metabolites, predominantly diterpenoids, along with alkaloids, terpenoids, flavonoids, and phenolic compounds [7].

*Croton macrostachyus* is widely recognized in Ethiopian traditional medicine for its therapeutic and insect‐repellent effects, particularly against mosquitoes. Its efficacy is attributed to the multiple bioactive compounds found in botanical extracts, which often work synergistically against ectoparasites through diverse modes of action. These include acetylcholinesterase inhibition, disruption of the octopaminergic system, and the induction of physiological stress via hydrophobic properties that impair water balance. This multitarget approach enhances parasiticidal efficacy while potentially delaying the development of resistance [8].

Plant‐based acaricides provide a sustainable, low‐resistance alternative to synthetic chemicals, offering environmental safety, zero livestock residues, and cost‐effective ectoparasites control with minimal nontarget risks [9]. However, limited research works are conducted in Ethiopia to exploit this potential. Hence, the present study was conducted to evaluate the acaricidal activity and synergistic effect of *Ricinus communis* seed and *Croton macrostachyus* leaf extracts against *Rhipicephalus-*major cattle tick and *Amblyomma*‐known tick for zoonotic pathogens transmissions.

## 2. Materials and Methodology

### 2.1. Study Area and Study Plants Authentication

The herbal plant species *Ricinus communis* (castor bean) and *Croton macrostachyus* were collected from Mante Dubo (substation) in the Areka Agricultural Research Center, Wolaita Zone, South Ethiopia. *Croton macrostachyus* and *Ricinus communis* were selected based on documented traditional use against ticks [10]. The plants were taxonomically identified and authenticated at the National Herbarium, Addis Ababa University, Ethiopia.

### 2.2. Plant Material Preparation and Extraction

Fresh leaves of *Croton macrostachyus* and seed of *Ricinus communis* were harvested during their flowering stage. A latex glove was used to prevent contamination and washed the leaves with distilled water to remove dirt and epiphytes. After cutting them into uniform pieces, the leaves were shade‐dried at room temperature for 2 weeks, protecting them from sunlight to preserve bioactive compounds. Once fully dried, the leaves were ground into a fine powder using a sterilized electric grinder and stored in airtight containers at 4°C until extraction.

Ethanolic extracts of *Croton macrostachyus* leaves and *Ricinus communis* seeds were prepared via cold maceration. For each species, 200 g of dried powder was soaked in 1500 mL of 70% ethanol in sealed glass containers and agitated for 72 h on an orbital shaker. The resulting crude extracts were first filtered through muslin cloth and then through Whatman No. 1 filter paper. Solvent removal was achieved using a rotary evaporator followed by complete drying in a temperature‐controlled oven. The concentrated extracts were stored in sterile amber glass bottles at 4°C to preserve heat‐sensitive compounds until use in bioassays [11].

### 2.3. Preliminary Screening of Phytochemical Constituents

Phytochemical screening of ethanol extracts from the *Ricinus communis* and *Croton macrostachyus* was conducted to identify secondary metabolites, revealing the presence of alkaloids via Mayer’s test indicated by turbidity from a precipitate, tannins through a blackish‐blue or brownish green coloration with ferric chloride, saponins confirmed by stable foam formation after vigorous shaking with distilled water, and flavonoids detected by a yellow coloration upon treatment with lead acetate solution. Moreover, the analysis was extended to phenols, where ethanol was added to the extracts and a portion of the resulting solution was warmed before adding ferric cyanide to produce a blue‐green color indicating polyphenols, while in a separate test, the formation of yellow precipitates upon the addition of lead acetate solution further confirmed the presence of phenolic compounds [12].

### 2.4. Tick Collection, Handling, and Identification

A total of 1080 viable adult ticks (540 *Rhipicephalus* spp. and 540 *Amblyomma* spp.) were collected from naturally infested cattle in Ethiopia’s Wolaita Zone. To ensure minimal stress to both ticks and host animals, ticks were gently removed from common attachment sites including the ears, udder, perianal region, and underside of the body using blunt forceps. Only fully engorged adult ticks were selected, with additional care taken to exclude those from cattle treated with acaricides within the past 3 months.

**FIGURE 1:**
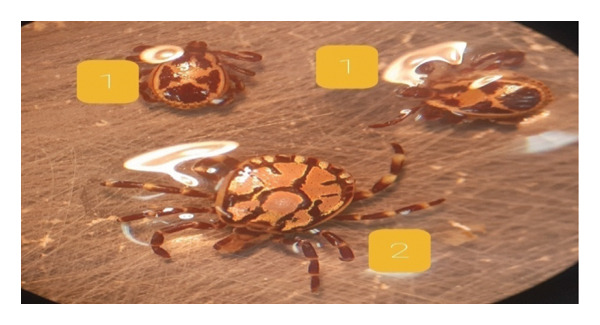
Representative adult ticks collected from the study area. (1) *Rhipicephalus spp.* and (2) *Amblyomma spp*.

Each collected tick was immediately placed in a labeled, ventilated container, recording key details such as the host animal’s sex, breed, age, collection date, location, and attachment site. The ticks were then transported to Addis Ababa University’s College of Veterinary Medicine (Department of Parasitology and Pathology) in insulated coolers to maintain optimal conditions. To ensure proper airflow while preventing escape, the containers were lined with cotton mesh. Before testing, all ticks were carefully examined under 10–40 × magnification and identified using established taxonomic keys [13]. Only active, undamaged ticks with normal behaviors—such as movement toward light and leg reflex responses—were included in the bioassays (Figure 1).

### 2.5. Experimental Design

This study investigated the acaricidal effects of ethanolic leaf extracts from *Croton macrostachyus* and *Ricinus communis*, both alone and in combination, against adult *Rhipicephalus* and *Amblyomma* ticks using the adult immersion test (AIT). The protocol for the combination aspect of the extracts was determined by 1:1. Four concentrations of each extract were prepared (125, 250, 500, and 1000 μg/mL) through serial dilution. Each concentration was tested in triplicate independent sets, with 10 ticks per group for each species.

For comparison, a positive control (0.1% diazinon, a known acaricide) and a negative control (2% DMSO, the solvent used for dilution) were used. Following the established method, the ticks were fully immersed in the treatment solutions for 5 min before transferring them to Petri dishes kept at 25°C [14]. Mortality was assessed at 30 min, 1, 2, and 4 h postexposure, with death confirmed by cessation of movement and darkening of cuticle coloration [15].

### 2.6. Mortality Percentage Rate

The mortality percentage rate of ticks exposed to extracts was calculated by the formula previously used [16].

(1) Mortality %=Total number of died ticksTotal number of exposed ticks×100.

The acaricidal efficacy of herbal extracts was classified based on mortality rates as very high (> 90% mortality), high (70%–90%), moderate (50%–70%), weak (30%–50%), and no efficacy (< 15%) [17].

### 2.7. Statistical Analysis

Mortality data were recorded in Excel and analyzed using SPSS 20. Results are presented as mean percentages ± standard error of mean (SEM). Treatment groups were compared using one‐way ANOVA with Tukey’s post hoc test for multiple comparison of acaricidal efficacy within and between treatment groups, considering *p* < 0.05 statistically significant.

## 3. Results

### 3.1. Extractive Value Determination and Bioactive Compound

The ethanolic extraction of 200 g dried plant material yielded 24.6 g (12.3%) crude extract from *Croton macrostachyus* and 22.8 g (11.4%) from *Ricinus communis* (Table 1). Phytochemical analysis revealed distinct bioactive profiles between the species (Table 2). *Croton macrostachyus* contained flavonoids, tannins, and phenols, while *Ricinus communis* showed alkaloids, tannins, saponins, and phenols.

**TABLE 1:** Percentage yield of herbals through hydroethanolic extraction.

Scientific name	Local name “Amharic”	Fine dried weight (gm)	Crude extract weight (gm)	Percentage yield (%)
*Ricinus communis* Delile	Gulo	200	22.8	11.4
*Croton macrostachyus* Linnaeus	Bisana	200	24.6	12.3

**TABLE 2:** Preliminary screening for phytochemical constituents.

Phytochemical constituents	*Croton macrostachyus*	*Ricinus communis*
Flavonoids	+	−
Alkaloids	−	+
Tannins	+	+
Saponins	−	+
Phenols	+	+

### 3.2. Acaricidal Efficacy of *Croton macrostachyus* and *Ricinus communis* Extracts

According to the present finding, the ethanolic extract of *Croton macrostachyus* demonstrated concentration‐dependent mortality against both tick species. The 1000 μg/mL concentration of *Croton macrostachyus* leaves extract exhibited 63.3 ± 1.16% mortality percentage rate in *Rhipicephalus ticks* after 4 h of exposure, which was significantly higher (*p* < 0.05) than lower concentrations and comparable to the positive control, diazinon (70.0 ± 1.00%). A similar trend was observed for *Amblyomma* ticks, where the 1000 μg/mL concentration resulted in 66.7 ± 1.53% mortality rate, which was not significantly different from diazinon (73.3 ± 1.16%) (Table 3 and Figure 2(a)).

**TABLE 3:** Acaricidal efficacy of *Croton macrostachyus* extracts against *Rhipicephalus* and *Amblyomma* ticks.

Conc. μg/mL	30 min	1 hr	2 hr	4 hr	30 min	1 hr	2 hr	4 hr
	*Rhipicephalus*				*Amblyomma*			
1000	1.33 ± 0.58^a^	2.67 ± 0.58^a^	4.33 ± 0.58^ab^	6.33 ± 1.15^a^	1.67 ± 0.58^a^	3.33 ± 0.58^a^	4.67 ± 0.58^ab^	6.67 ± 1.53^ab^
500	0.67 ± 0.58^ab^	1.33 ± 0.58^ab^	2.67 ± 0.58^bc^	3.33 ± 0.58^b^	1.00 ± 0.00^ab^	2.67 ± 0.58^ab^	3.33 ± 0.58^bc^	4.33 ± 0.58^bc^
250	0.33 ± 0.58^ab^	1.00 ± 0.00^b^	1.67 ± 0.58^cd^	2.33 ± 1.53^bc^	0.67 ± 0.58^ab^	1.33 ± 0.58^bc^	2.00 ± 0.00^cd^	2.67 ± 0.58^c^
125	0.00 ± 0.00^b^	0.33 ± 0.58^b^	1.00 ± 1.00^cd^	2.00 ± 1.00^bc^	0.33 ± 0.58^b^	0.67 ± 0.58^c^	1.33 ± 0.58^de^	2.33 ± 0.57^cd^
2% DMSO	0.00 ± 0.00^b^	0.00 ± 0.00^b^	0.00 ± 0.00^d^	0.00 ± 0.00^c^	0.00 ± 0.00^b^	0.00 ± 0.00^c^	0.00 ± 0.00^e^	0.00 ± 0.00^d^
0.1% Diazinon	0.00 ± 0.00^b^	1.00 ± 1.00^b^	5.00 ± 1.00^a^	7.00 ± 1.00^a^	0.33 ± 0.58^b^	1.33 ± 0.58^bc^	5.33 ± 1.15^a^	7.33 ± 1.16^a^
G.Mean	0.39	1.06	2.44	3.50	0.67	1.56	2.78	3.89
SEM	0.14	0.24	0.45	0.63	0.16	0.29	0.47	0.64

*Note:* Mean mortality values with different subscripts in the same column are significantly different (*p* < 0.05). DMSO: dimethyl sulfoxide, G.Mean: geometric means, SEM: standard error of the mean.

FIGURE 2Acaricidal activity of synergism, ricinus, and croton (a, b, and c), respectively.

**Figure:**
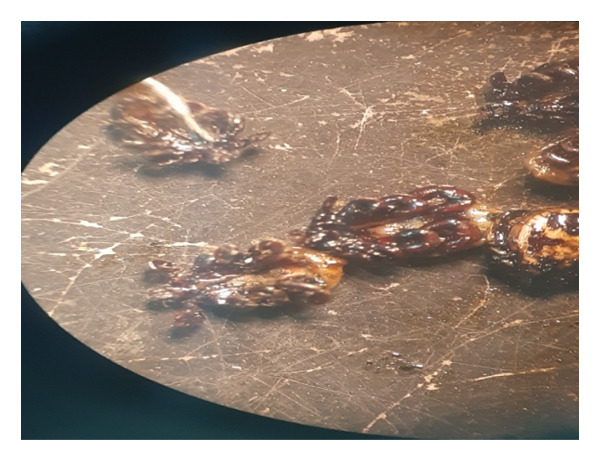
(a)

**Figure:**
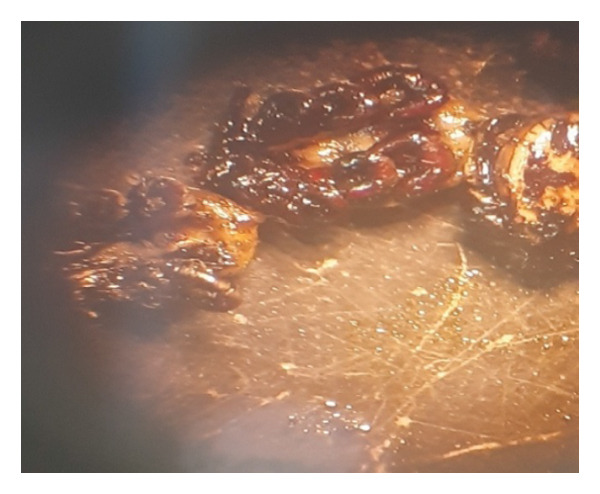
(b)

**Figure:**
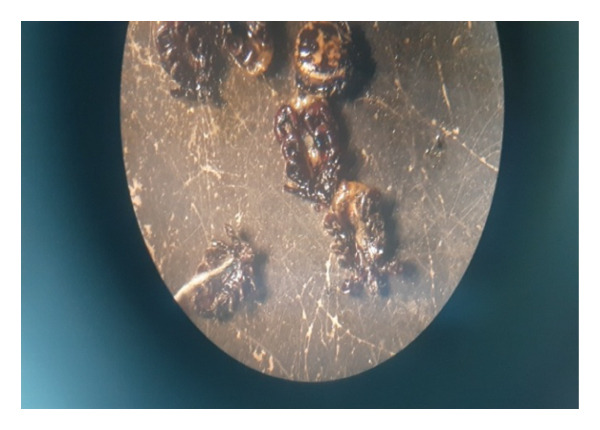
(c)

Notably, the *Ricinus communis* extract demonstrated significant acaricidal activity, achieving 73.3 ± 0.58% mortality percentage rate in *Rhipicephalus spp.* and 80.0 ± 1.00% in *Amblyomma spp*. *Ricinus communis* was significantly more effective against *Amblyomma spp.* than the reference drug diazinon (73.3 ± 1.15% mortality rate, *p* < 0.05). Its activity against *Rhipicephalus spp.* was comparable to diazinon (70.0 ± 1.00%, *p* > 0.05) (Table 4 and Figure 2(b)).

**TABLE 4:** Acaricidal efficacy of *Ricinus communis* extracts against *Rhipicephalus* and *Amblyomma* ticks.

Conc. μg/mL	30 min	1 hr	2 hr	4 hr	30 min	1 hr	2 hr	4 hr
	*Rhipicephalus*				*Amblyomma*			
1000	1.33 ± 0.58^a^	2.33 ± 0.58^a^	4.00 ± 1.00^ab^	7.33 ± 0.58^a^	1.67 ± 1.15	2.33 ± 0.58^a^	6.33 ± 0.58^a^	8.00 ± 1.00^a^
500	1.00 ± 0.00^ab^	2.00 ± 0.00^ab^	2.67 ± 0.58^b^	4.33 ± 0.58^b^	1.33 ± 0.58	2.00 ± 0.00^ab^	3.33 ± 0.58^b^	5.00 ± 1.00^b^
250	0.67 ± 0.58^ab^	1.00 ± 0.00^bcd^	2.33 ± 0.58^b^	3.00 ± 0.00^bc^	1.00 ± 0.00	1.00 ± 0.00^bcd^	2.33 ± 0.58^b^	3.33 ± 0.58^bc^
125	0.33 ± 0.58^ab^	1.33 ± 0.58^abc^	2.00 ± 1.00^bc^	2.33 ± 0.58^c^	0.67 ± 0.58	1.33 ± 0.58^abc^	1.67 ± 0.58^bc^	2.67 ± 0.58^c^
2% DMSO	0.00 ± 0.00^b^	0.00 ± 0.00^d^	0.00 ± 0.00^c^	0.00 ± 0.00^d^	0.00 ± 0.00	0.00 ± 0.00^d^	0.00 ± 0.00^c^	0.00 ± 0.00^d^
0.1% Diazinon	0.00 ± 0.00^b^	0.67 ± 0.58^cd^	5.00 ± 1.00^a^	7.00 ± 1.00^a^	0.33 ± 0.58	0.67 ± 0.58^cd^	5.33 ± 1.16^a^	7.33 ± 1.15^a^
G.Mean	0.56	1.22	2.67	4.00	0.83	1.22	3.17	4.39
SEM	0.15	0.21	0.41	0.63	0.19	0.21	0.54	0.69

*Note:* Mean mortality values with different subscripts in the same column are significantly different (*p* < 0.05). DMSO: dimethyl sulfoxide, G.Mean: geometric means, SEM: standard error of the mean.

### 3.3. Synergistic Acaricidal Activities of Combined Extracts

The combination of *Croton macrostachyus* and *Ricinus communis* extracts demonstrated significantly greater acaricidal activity against both *Amblyomma* and *Rhipicephalus* species compared with either extract used alone (*p* < 0.05) (Table 5). The combined extracts demonstrated enhanced acaricidal activity, particularly against *Amblyomma* (Figure 2(c)). The combined formulation of extract (1000 μg/mL) demonstrated significantly higher mortality rate (*p* < 0.05) than both individual extracts and synthetic acaricide, achieving 83.3 ± 0.58 and 76.7 ± 0.58% mortality against *Amblyomma* and *Rhipicephalus* ticks, respectively.

**TABLE 5:** Synergistic efficacy of extracts against *Rhipicephalus* and *Amblyomma*.

Conc. μg/mL	30 min	1 hr	2 hr	4 hr	30 min	1 hr	2 hr	4 hr
	*Rhipicephalus*				*Amblyomma*			
1000	1.00 ± 1.00	2.33 ± 0.58^a^	4.67 ± 2.08^a^	7.67 ± 0.58^a^	2.00 ± 1.00^a^	4.67 ± 0.58^a^	6.33 ± 0.58^a^	8.33 ± 0.57^a^
500	0.67 ± 0.58	1.67 ± 0.58^ab^	3.67 ± 1.53^ab^	5.00 ± 1.00^bc^	1.33 ± 0.57^ab^	3.33 ± 0.58^ab^	4.33 ± 0.58^ab^	6.67 ± 0.57^a^
250	0.33 ± 0.58	1.33 ± 0.58^abc^	2.67 ± 0.558^abc^	4.00 ± 1.00^cd^	0.67 ± 0.57^ab^	2.00 ± 1.00^bc^	3.00 ± 1.00^bc^	4.67 ± 0.57^b^
125	0.00 ± 0.00	0.33 ± 0.58^bc^	1.33 ± 0.58^bc^	2.33 ± 0.58^d^	0.33 ± 0.58^ab^	1.33 ± 0.58^bc^	1.67 ± 0.58^cd^	2.67 ± 0.57^c^
2% DMSO	0.00 ± 0.00	0.00 ± 0.00^c^	0.00 ± 0.00^c^	0.00 ± 0.00^e^	0.00 ± 0.00^b^	0.00 ± 0.00^c^	0.00 ± 0.00^d^	0.00 ± 0.00^d^
0.1% Diazinon	0.00 ± 0.00	1.33 ± 0.58^abc^	5.00 ± 1.00^a^	7.00 ± 1.00^ab^	0.33 ± 0.58^ab^	1.67 ± 1.15^bc^	5.33 ± 1.16^a^	7.33 ± 1.15^a^
G.Mean	0.33	1.17	2.89	4.33	0.78	2.17	3.44	4.97
SEM	0.14	0.22	0.49	0.66	0.21	0.39	0.54	0.71

*Note:* Mean mortality values with different subscripts in the same column are significantly different (*p* < 0.05). DMSO: dimethyl sulfoxide, G.Mean: geometric means, SEM: standard error of the mean.

## 4. Discussion

The study revealed the crude extractive values of *Croton macrostachyus* (12.3%) and *Ricinus communis* (11.4%) that were lower than the finding reported for *Croton macrostachyus* (29.3%) and *Ricinus communis* (30.67%) by [18]. The observed difference in extraction yields may be attributed to several methodological and biological factors. Key variables likely influencing these differences include variations in plant age, agroecological conditions, solvent polarity, and extraction efficiency.

The study confirmed the presence of phenolic compounds, flavonoids, and saponins in *Croton macrostachyus*, contradicting the findings that showed no flavonoids in their phytochemical analysis based on [18]. In contrast, *Ricinus communis* contains phenolics, tannins, saponins, and alkaloids. These results are consistent with earlier reports by [18, 19]. However, this report contradicts the study finding that indicated none of these compounds detection according to [20]. These variations are likely due to variations in extraction solvents and testing protocols, as noted by highlighting the influence of methodological differences on phytochemical detection [21].

The study finding demonstrated significant acaricidal activity of both plant extracts against *Amblyomma* and *Rhipicephalus* ticks, with mortality rates exhibiting concentration‐ and time‐dependent increases. These findings align with the study result report confirming that acaricidal efficacy is dose and duration dependent [22].

The study found that *Croton macrostachyus* leaf extract exhibited only moderate acaricidal activity against *Amblyomma* (66.7 ± 1.53% mortality) and *Rhipicephalus* (63.3 ± 1.16% mortality) ticks, even at high concentrations (1000 μg/mL) and prolonged exposure times (4 h). These results contrast with the findings that reported very high (> 90%) acaricidal activity of *Croton macrostachyus* [18]. The observed difference may be attributed due to phytochemical variations and methodological factors in testing protocols.

The ethanoic extract of *Ricinus communis* exhibited high acaricidal activity against *Amblyomma* (80.0 ± 1.00% mortality) and *Rhipicephalus* (73.3 ± 0.58% mortality) ticks at 1000 μg/mL. These results are consistent with the finding that reported comparable acaricidal activity against *Rhipicephalus* [23]. However, the present finding was inconsistent with the report indicated very high activity (≥ 90% mortality) using a methanolic extract at a concentration of 200 mg/mL, suggesting that extraction solvent and dose of extract significantly influences acaricidal activity [17].

Notably, the finding demonstrated that the combined C*roton macrostachyus* and *Ricinus communis* extracts showed significantly higher mortality rate of ticks than individual extracts or diazinon, and the activity was dose and time dependent. The demonstrated synergy indicates that the combined phytochemical profile of both plants creates a more comprehensive acaricidal effect than either extract alone.

## 5. Conclusion and Recommendations

The extracts of *Croton macrostachyus* and *Ricinus communis* demonstrated substantial acaricidal activity, with the combined formulation showing synergistic effects. These botanical extracts showed a clear dose‐ and time‐dependent relationship in their tickicidal action. The presence of bioactive phytochemicals likely contributes to this observed activity, indicating these plant extracts as promising, environmentally sustainable alternatives to synthetic tick control methods.

## Author Contributions

Tesfaye Fatalo: conceptualization, designing methodology, field data collection, supervision of field trial, data analysis, and original draft writing. Gebeyehu Alkadir: laboratory works.

## Funding

This study was funded by Southern Agricultural Research Institute, Ethiopia.

## Conflicts of Interest

The authors declare no conflicts of interest.

## Data Availability

The data that support the findings of this study are available from the corresponding author upon reasonable request.
